# Air Manganese Levels and Chronic Liver Disease Mortality in North Carolina Counties: An Ecological Study

**DOI:** 10.3390/ijerph9093258

**Published:** 2012-09-05

**Authors:** John G. Spangler

**Affiliations:** Department of Family and Community Medicine, Wake Forest School of Medicine, Winston-Salem, NC 27157, USA; Email: jspangle@wakehealth.edu; Tel.: +1-336-716-2238; Fax: +1-336-716-1297

**Keywords:** manganese, air pollution, liver disease, ecological study

## Abstract

A Manganese is an essential trace element which is toxic in high doses. Over the past several decades, manganese has replaced lead as the anti-knock agent in gasoline, raising concern about air and road-side contamination with this element. In addition, manganese is absorbed by the liver, making specific populations (e.g., pregnant women, infants and children, and patients with liver disease) susceptible to its toxic effects. Using data from the US Census Bureau, the North Carolina State Center for Health Statistics, and the US Environmental Protection Agency, this ecological study evaluated chronic liver disease mortality rates in North Carolina’s 100 counties. It correlated these rates with county-level demographics as well as on-road and non-road air borne manganese concentrations. Median income by county was inversely associated with chronic liver disease mortality, while the logarithmically transformed airborne concentrations of on-road manganese were positively correlated with county-level chronic liver disease mortality. Because environmental manganese near roads is likely to increase over time, these pilot findings potentially have regulatory implications and argue for further research.

## 1. Introduction

The trace element, manganese, is essential to health but toxic in high doses [1]. In addition to neurotoxicity, manganese also damages the liver. Because manganese is absorbed and excreted by the liver, it is a target organ for toxicity [1], likely through free radical generation in the mitochondria [2]. In a review of the effects of manganese on mitochondria Gunter *et al*. [2] noted that this element “decreases energy metabolism *in vivo *and *in vitro*, including decreases in the activities of mitochondrial enzymes, in membrane potential, and ATP production” which may thereby damage tissue. Manganese accumulation in the mitochondria, including liver mitochondria, also inhibits oxidative phosphorylation [3]. In rats, manganese-mitochondria interaction increases the production of the [OH] hydroxyl radical [4]. In addition, manganese overload is commonly found among patients with chronic liver failure, and presents clinically in a similar fashion as chronic liver disease with similar imaging and pathological findings [4]. Often, there is also similar neurologic dysfunction in liver failure, which has been attributed to excessive deposition of this element in the basal ganglia [5]. Indeed, excessive liver storage of manganese might contribute to liver fibrosis in the cirrhotic liver [6]. Because manganese shares absorption and excretion mechanisms with iron, both iron overload [6] and iron deficiency [7,8] make the liver susceptible to manganese toxicity.

Since manganese is processed by the liver, diseases of the liver may increase storage and therefore toxicity of this metal which could be reflected Chronic liver disease mortality in North Carolina is slightly higher than that of the US nationally (9.2 *vs*. 9.1 per 100,000 population) [9]. The 2005 Behavioral Risk Factor Surveillance Survey for North Carolina showed that 25% of residents drank five or more drinks on at least one occasion during the past 30 days; and 10% drank five or more drinks on three or more occasions over the past 30 days [10]. Nationally, only 14% of Americans drank 5 or more drinks on one occasion during the past 30 days [11]. Thus, it stands to reason that liver disease, if not liver mortality, is higher in North Carolina compared to the rest of the nation. Given this information, the contribution of manganese to liver mortality is an important issue to consider for public policy regarding population health in the state.

This is all the more urgent since manganese has replaced lead as an octane booster in gasoline worldwide in the form of methylcyclopentadienyl manganese tricarbonyl (MMT) [12]. Thus, investigation into the potential link between manganese and adverse health outcomes is particularly important because exposure to environmental sources of this element will likely increase in the coming years [12]. Exposure to manganese in car exhaust would likely be highest near roads, such that air concentrations of on-road manganese would be the most predictive at the population level of this element’s contribution to liver disease [13].

## 2. Methods

County level data were derived from the 2000 US Census US Census Bureau [14] and included total county population, total county urban and rural population, median income of counties, poverty rate, and the percent of the counties’ population without a high school education. Using the North Carolina Center for State Health Statistics data combined for years 1997–2001, mortality from chronic liver disease was derived by county [15].

Average air on-road and non-road manganese levels by county (mcg/m^3^), as reported by the 1999 national air toxic assessment by the US Environmental Protection Agency (EPA) [16] were also gathered. 

Manganese concentrations were logarithmically transformed (to the base 10) to provide a normal distribution. Stepwise multiple regression was then carried out using liver mortality as the dependent variable and manganese and county factors as independent variables. Data were entered in SPSS version 16; significance was set at *P* < 0.05.

## 3. Results

County level variables are listed in Table 1. 

**Table 1:** North Carolina county-level variables of demographic, liver mortality and manganese air concentrations on and off roads.

County Variables	Median	Range
Population	47,879	4,150–695,450
Median Income	$34,013	$25,180–$54,990
Liver disease mortality per 100,000 population	9.25	0–23.2
Log (On-road Mn) * mg/m^3^	−5.404	−7.280 to −4.640
Log (Non-road Mn) * mg/m^3^	−6.429	−7.06 to −5.10

* Logarithm of the concentrations of airborne manganese on and off roads, originally measured in mg/m^3^.

Median population of counties was approximately 48,000, with median income of about $34,000. Median liver disease mortality per 100,000 population was 9.25. The mean log of the concentration of on-road manganese was −5.404 (range −7.280 to −4.640, about 2.6 orders of magnitude); log concentration of off-road manganese was −6.429 (range −7.060 to −5.100, approximately 2 orders of magnitude). Stepwise multivariable regression (Table 2) indicates that liver mortality is inversely proportional to median county income (in thousands of dollars); and to the log of the median concentration of on-road manganese. 

**Table 2:** Multivariable regression of county level correlates of chronic liver disease mortality in North Carolina Counties (n = 100).

Model	B	Standard Error	*P* value
County Median Income	−0.326	0.071	<0.001
Log (On-road Mn) * mg/m^3^	3.167	0.865	<0.001

* Logarithm of the concentrations of airborne manganese on roads, originally measured in mg/m^3^.

## 4. Discussion and Conclusions

This study has found that death from chronic liver disease, including cirrhosis, on the county level in North Carolina’s 100 counties correlates with on-road concentrations of manganese. Because manganese is an additive to gasoline in many parts of the world [12], including North Carolina, car exhaust is likely the major source of on-road airborne manganese [13]. While regulation of manganese in gasoline—or regulation of manganese air concentrations—would require major public policy changes, the issue is worth considering. This is because patients with chronic liver disease are not the only population vulnerable to environmental manganese exposure. Pregnant women, who have high iron requirements, absorb manganese more efficiently than non-pregnant women, exposing themselves and the fetus, to potentially toxic manganese accumulation [1]. Such exposure can affect infant development [17]. In addition, environmental manganese exposure (through drinking water) has been shown to correlate with infant mortality in Bangladesh [18] as well as in North Carolina [19]. Also, manganese has been shown to have other adverse neurodevelopmental effects on children, including hyperactive behavior [20] and poorer academic achievement [21]. Adults may also experience toxic effects from manganese exposure, such as the development of manganism (a condition similar to Parkinson’s disease) [1]; violent and aberrant behavior [22]; and other neurotoxic effects [23,24,25].

The association between lower median county income levels and chronic liver disease mortality might be explained by the known increase in alcohol related mortality and lower socioeconomic status [26,27]. Heidelbaugh and Bruderly [28] review the evidence regarding chronic liver disease and cirrhosis mortality. Up to 70% of cirrhosis cases and up to 80% of chronic liver disease death are related to alcohol use. While frequency of alcohol consumption is increased in higher socioeconomic groups, problem drinking, drinking to intoxication and alcohol death are more strongly correlated to lower socioeconomic status [26]. Indeed, lower education, unemployment, reduced personal income and lower net household income all increase the risk of acute and chronic alcohol related deaths [26].

The findings of this study are subject to a number of limitations. First, this study employed an ecological analysis which matches disease patterns in populations with risk factors at the population level [29]. Because analyses are population based, they cannot be extended down to the individual level, e.g., on cannot say that any specific individual exposed to these levels of on-road manganese will die from chronic liver disease. In addition, ecological studies are best utilized for hypothesis generation to guide future studies based on findings at the population level [29]. They cannot control for confounding of known or unknown factors related to chronic liver disease mortality. In addition, this study would be greatly strengthened if alcohol consumption levels were known at the county level. Unfortunately, in North Carolina, those data do not exist for all 100 counties. Also, the toxicity of manganese is dependent on its species and chemical formula, since various manganese compounds have different solubility and bioavailability. Such information is not available from the US EPA. Furthermore, the compounds emitted in car exhaust might not be the specific manganese compounds that correlate with liver disease mortality, even though the on-road measurement of manganese compounds strongly hints that this is the case. Finally, this study’s “exposure” to manganese (e.g., EPA data from 1999) [16] predated measured county-level mortality (2001 data) [15]. While this temporality of effect might support generating a hypothesis of causality, cross sectional data cannot be used to prove causality. 

Nonetheless, these findings have implications not only for future research, but also for public policy. Given the presence of manganese in gasoline, policy making and regulatory agencies might be wise to err on the side of caution by controlling the amount of MMT in automotive fuel or at least by regulating manganese airborne levels.
